# Epidermal and Dermal T Cells Exhibit Distinct Proteomic Signatures

**DOI:** 10.3390/ijms26167942

**Published:** 2025-08-18

**Authors:** Amalie Arvesen, Marcel B. M. Teunissen, Sofie Agerbæk, Bjørn Kromann, Line Bruun Pilgaard Møller, Ahmed Gehad, Rachael A. Clark, Marianne Bengtson Løvendorf, Beatrice Dyring-Andersen

**Affiliations:** 1 Department of Dermatology, Zealand University Hospital, 4600 Roskilde, Denmarksoage@regionsjaelland.dk (S.A.); bjhan@regionsjaelland.dk (B.K.);; 2 Department of Dermatology, Amsterdam Institute for Immunology and Infectious Diseases, Amsterdam University Medical Centers, Location AMC, 1105 AZ Amsterdam, The Netherlands; m.b.teunissen@amsterdamumc.nl; 3 Department of Dermatology and Allergy, Copenhagen University Hospital-Herlev and Gentofte, 2900 Hellerup, Denmark; 4 Department of Dermatology, Mass General Hospital, Harvard Medical School, Boston, MA 02115, USA; agehad@bwh.harvard.edu (A.G.); rclark@bwh.harvard.edu (R.A.C.); 5 The Leo Foundation Skin Immunology Research Center, Faculty of Health and Medical Sciences, University of Copenhagen, 1172 København, Denmark; 6 Novo Nordisk Foundation Center for Protein Research, Faculty of Health and Medical Sciences, University of Copenhagen, 1172 København, Denmark

**Keywords:** T cells, skin, epidermis, dermis, immune cells, adaptive immunity, HLA, proteomics

## Abstract

T lymphocytes in human skin play essential roles in immune surveillance and tissue homeostasis, with distinct populations residing in the epidermal and dermal compartments. To characterize the molecular basis of their compartmentalized functional specialization, we performed proteomic analysis of total T cell populations isolated from healthy human skin, combining flow cytometry and liquid chromatography–tandem mass spectrometry. We quantified 5985 proteins across epidermal and dermal T cell populations, identifying 2177 significantly differentially expressed proteins (FDR < 0.05), including 1008 with >2-fold changes. Compared with dermal T cells, epidermal T cells showed elevated intensity of tissueresidency marker CD69, co-stimulatory protein CD27, complement components (C3, C4a, and Factors B and D), and proteins involved in oxidative phosphorylation and cholesterol metabolism. Epidermal T cells also exhibited higher levels of antimicrobial S100 proteins, chemokine receptor CCR6, IL-18, and MHC class I molecules, while, in contrast, dermal T cells showed increased expression of CXCR4, IL-16, and MHC class II-related proteins. While these distinct proteomic signatures suggest compartment-specific adaptations in metabolism, immune surveillance, and antigen presentation, the results should be interpreted as exploratory, given methodological limitations. Nonetheless, this study provides a valuable molecular resource for understanding the specialization of T cells within different skin layers and offers a basis for future investigations into skin immune biology and its potential implications in disease.

## 1. Introduction

The skin serves as an important barrier organ, harboring diverse populations of immune cells that maintain tissue homeostasis and provide protection against pathogens. T lymphocytes are essential components of this immune surveillance system, with distinct populations residing in both the epidermal and dermal compartments. These tissue-resident T cells play crucial roles in immediate immune responses and long-term immunological memory [1,2].

The distribution and phenotypic characteristics of T cells differ between the epidermal and dermal layers, reflecting distinct microenvironmental challenges and functional requirements. While CD4+ and CD8+ T cells coexist in both compartments, CD4+ T cells are more prevalent in the dermis, whereas CD8+ T cells predominate in the epidermis, suggesting functional specialization between these layers^3^. However, beyond this binary classification, cutaneous T cells comprise diverse subsets, including tissue-resident memory T (Trm) cells, mucosal-associated invariant T (MAIT) cells, regulatory T (Treg) cells, and γδ T cells, each contributing uniquely to local immune responses [3,4,5,6]. Furthermore, the categorization of T cells into functionally distinct subsets, such as type 1 helper T cells (Th1), Th2, and Th17, as well as developmentally distinct subsets, including naïve, central memory, and effector memory T cells, adds additional layers of complexity. Given this heterogeneity, our study aimed to characterize the overall differences between total dermal and total epidermal T cell populations, rather than focusing on individual T cell subsets. Specifically, we sought to identify global proteomic differences related to homing properties, metabolic adaptations, and immune regulatory pathways between these two compartments. For example, T cell function is intricately linked to their metabolic program, and tissue-resident memory T cells have been shown to utilize specific metabolic pathways for their long-term maintenance in tissues [7,8]. However, despite these emerging insights, the skin layer-specific differences in skin T cells remain incompletely analyzed and quantified, thus preventing a deeper understanding of the molecular basis for their functional differences.

Recent advances in mass spectrometry-based proteomics have enabled detailed characterization of cellular proteomes, providing insights into functional specialization and regulatory mechanisms [9,10,11,12]. This method offers a powerful approach to comprehensively profile the protein expression patterns in immune cells, shedding light on their activation states, functional capabilities, and interactions with the surrounding microenvironment. While previous studies, including our own, have examined T cell populations in the epidermis and dermis, continuous improvements in proteomics technology and database curation have substantially increased protein detection sensitivity and depth [9,13].

In this study, we leveraged high-resolution mass spectrometry combined with updated proteomic databases and quantification algorithms to achieve a far more comprehensive characterization of the epidermal and dermal T cell proteomes than previously possible. This approach enabled the identification of novel protein signatures related to metabolism, complement pathway components, and immune regulatory mechanisms between these populations. While differences in T cell subset composition may contribute to some of the observed variations in this study, our proteomic approach provides insights that transcend conventional subset classifications, offering a broader, data-driven perspective on T cell compartmentalization within the human skin.

## 2. Results

### 2.1. Proteomic Analysis Revealed Distinct Protein Signatures in Epidermal and Dermal T Cells

**Figure 1:**
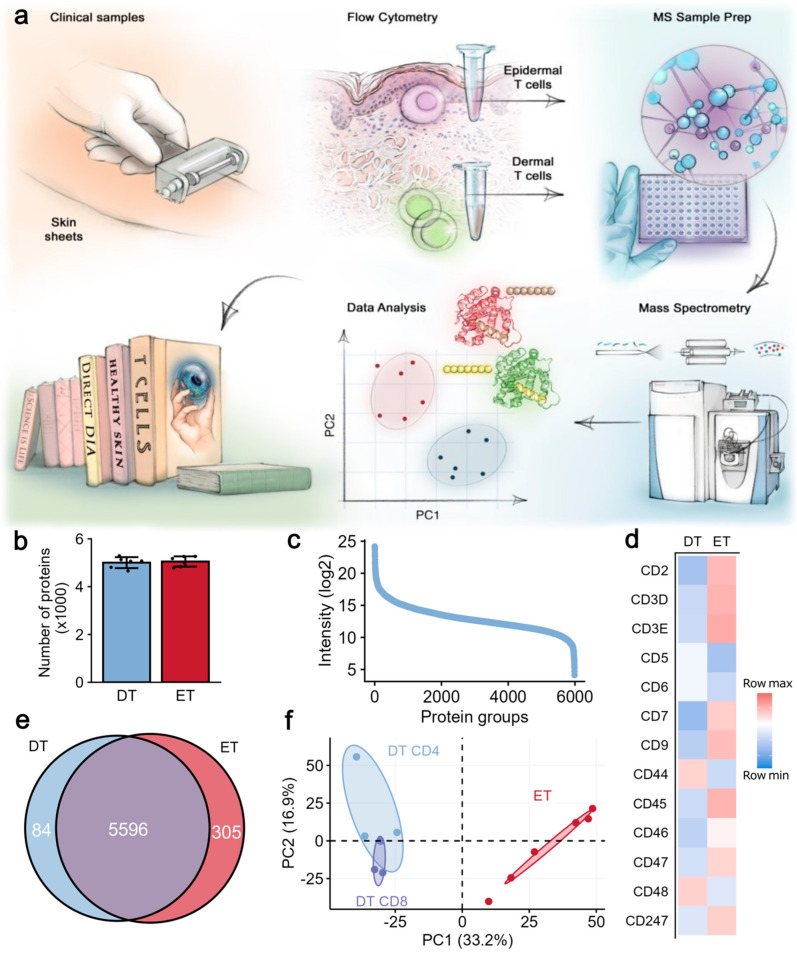
**Quantitative, spatially resolved proteomic analysis of T cells from different skin layers.** (**a**) Epidermis and dermis of healthy skin were separated enzymatically, and cells from these two layers were labeled with fluorochrome-conjugated antibodies and sorted using flow cytometry. Sorted cells underwent sample preparation for LC-MS/MS and were analyzed in data-independent acquisition (DIA) mode. Mass spectrometry data were analyzed with a hybrid search that included a fractionated PBMC library. (**b**) Number of protein groups quantified in ETs and DTs from each donor. A total of 4688 proteins were quantified. (**c**) Protein ranks of mean intensities of all samples. (**d**) Heatmap colored by row Z-scores of intensities of selected CD genes in DTs and ETs. (**e**) Venn diagram depicting ET and DT proteomes, comprising, in total, 5985 proteins. (**f**) Principal component analysis of all proteomes from ETs (red dots) and DTs (blue dots). CD4 and CD8 subsets in DT samples are labeled. PBMCs = peripheral blood mononuclear cells, ETs = epidermal T cells, DTs = dermal T cells, and CD = cluster of differentiation.

We investigated protein expression differences between CD3+ T cells from the epidermis (ETs; N = 6) and total CD3+ T cells from the dermis (DTs; N = 3), the latter comprising paired purified CD4^+^ and CD8^+^ subsets, as previously published as part of the Human Skin Proteome Atlas [9]. Skin T cells were isolated by flow cytometry, followed by liquid chromatography–tandem mass spectrometry (LC-MS/MS) (Figure 1a). While DTs were originally separated into CD4^+^ and CD8^+^ subsets, for this study, they were combined into a total DT population to enable comparison with ETs, which were isolated as a single CD3^+^ population (without separation into CD4^+^ and CD8^+^ T cells) to ensure sufficient yield for in-depth proteomic analysis (Supplementary Figure S1a,b). This strategy, while allowing sufficient cell numbers and depth of coverage, introduced limitations, such as subset mixing, and precluded direct CD4/CD8 comparisons within the epidermal compartment. The fast-paced technological developments enabled us to re-analyze the mass spectrometry raw files and quantify 5985 protein groups in total (between 4684 and 5651 protein groups), amounting to an average of 39% more proteins per sample compared with the previous publication (Figure 1b and Supplementary Tables S1 and S2). We filtered the dataset for proteins quantified in at least two-thirds of the samples in at least one group, with 5604 proteins remaining for downstream analyses (amounting to an average of 57% more proteins/sample than previously published; Supplementary Table S3). The intensities spanned five orders of magnitude (Figure 1c) and included, as expected, a range of T cell-defining markers, such as CD3, CD247, and T cell-associated proteins CD2, CD5, CD6, CD9, CD44, CD45 (encoded by the *PTPRC* gene), and CD47 (Figure 1d). CD4, CD8α, and CD7 were identified in a subset of samples (Supplementary Figure S1c). Most proteins were quantified in both ET and DT proteomes (5596 proteins (93.5%); Figure 1e). We analyzed the transcription factors present in cutaneous T cells and found 372 known transcription factors, including those associated with functionally distinct T cell subsets, such as T-bet (encoded by *TBX21*), GATA3, RORγt (*RORC*), AHR, and FOXP3 (Supplementary Table S4). Principal component analysis showed grouped cellular subsets, suggesting differences between the ETs and DTs and relative homogeneity within the groups (Figure 1f). Taken together, we reanalyzed proteomes of T cells derived from the epidermis and dermis of healthy human skin and observed that, although most proteins were present in both the epidermal and dermal T cells, the two T cell populations also exhibited distinguishable signatures. These results should be interpreted as exploratory due to pooled sampling, enzymatic dissociation differences, and unseparated T cell subsets in the ET population.

### 2.2. Pathway Enrichment Analysis Highlights Distinct Metabolic Profiles in Epidermal and Dermal T Cells

**Figure 2:**
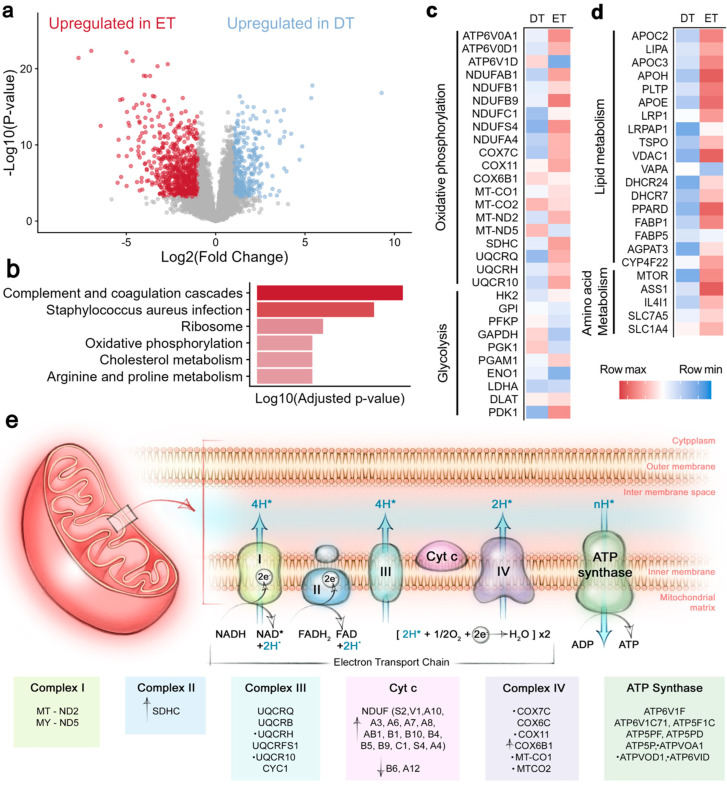
**Analysis of differentially expressed proteins and pathway enrichment.** (**a**) Volcano plot of differential expression analysis. Proteins with fold change > 2 and FDR < 5% are colored. (**b**) Pathway enrichment analysis of differentially expressed proteins between ETs and DTs (KEGG). (**c**) Heatmap colored by row Z-score of selected proteins involved in oxidative phosphorylation, glycolysis, and lipid and amino acid metabolism. (**d**) Schematic illustration of oxidative phosphorylation pathway. Dots indicate differentially expressed proteins with FCH > 2, and arrows indicate if the proteins were upregulated or downregulated in ETs versus DTs. (**e**) Differentially expressed proteins involving all major steps of oxidative phosphorylation (OXPHOS) in ETs compared with DTs. Proteins marked by a dot have a log-2 fold change >1. FDR = false discovery rate, ETs = epidermal T cells, and DTs = dermal T cells.

To understand these distinct features, we next applied a differential expression analysis of the T cells from the epidermal and dermal compartments. The analysis revealed 2177 proteins that were significantly differentially expressed between ETs and DTs (FDR < 0.05), including 1008 proteins with a minimum fold change (FCH) of two (Figure 2a and Supplementary Table S3). Pathway enrichment analysis highlighted that differentially expressed proteins were associated with cholesterol metabolism, oxidative phosphorylation, and complement pathways (Figure 2b).

We found higher intensities of the proteins involving all major steps of oxidative phosphorylation (OXPHOS) in ETs compared with DTs (Figure 2c,e), including PDK1 (FCH = 4.35), the enzyme that inhibits conversion of pyruvate to acetyl-CoA as part of aerobic glycolysis. Conversely, in DTs, we noted significantly higher intensities of the glycolysis pathway enzymes GAPDH (FCH = 1.77), which catalyzes the conversion of glyceraldehyde 3-phosphate to d-glycerate 1,3-bisphosphate, and DLAT (FCH = 1.54), which links glycolysis to the tricarboxylic acid (TCA) cycle.

We also noted differences involving lipid metabolism between ETs and DTs. The epidermis is an active site for cholesterol synthesis, which is essential for the integrity of the skin barrier. Consequently, the cellular environment and available nutrients of ETs and DTs presumably differ. ETs exhibited significantly higher intensities of several apolipoproteins (APOC2, APOC3, APOE, and APOH) (FCH = 31.4, 6.94, 3.27, and 4.72, respectively) and TSPO (FCH = 2.05), a protein that facilitates cholesterol transport across the mitochondrial membrane. We also found significantly higher levels of DHCR24 (FCH = 6.13) and DHCR7 (FCH = 1.56), both involved in the biosynthesis of cholesterol, in ETs (Figure 2d and Supplementary Table S3).

Additionally, PPARd (FCH = 8.55), the only PPAR protein identified in this dataset from its lipid transport family, had significantly higher intensity in ETs. We found no differences between ETs and DTs in the fatty acid transport proteins FABP1, FABP5, or SLA27A1-4. FAB4 and CD36 were not identified. We observed higher quantities of enzymes that are required for the production of the triacylglycerides ACSL1 (FCH = 2.15) and AGPAT3 (FCH = 1.95) in ETs. Moreover, AGPAT1/2/4 were quantified in ETs but not in DTs. We found similar intensities of lysosomal acid lipase (LIPA), which hydrolyzes triacylglycerides (TAGs) to support mitochondrial FAO, in both groups (Figure 2d and Supplementary Table S3). CYP4F22 (FCH = 3.13), a member of the cytochrome P450 family, involved in the metabolism of lipids with a known role in the skin [14], was higher in ETs compared with DTs.

In addition to the changes in lipid metabolism, we observed changes in molecules regulating amino acid transport. Notably, ETs expressed higher levels of mTOR (FCH = 1.49) and SLC7A5 (FCH = 8.48) (Figure 2d and Supplementary Table S3). SLC7A5, in combination with SLC3A2, forms an amino acid transporter, Lat1, that activates mTOR by exchanging leucine for glutamine to increase intracellular leucine levels [15], and our data indicated higher levels of transport in ETs. Meanwhile, we found no difference in alanine/serine/cysteine/glutamine transporter SLC1A5 (ASCT2), a solute carrier whose expression increases after T cell activation [16].

Arginine plays an important role in regulating the metabolic program of T cells [17]. Here, we found a marked difference in the expression of ASS1 (FCH = 9.35), which regulates arginine metabolism and is involved in converting citrulline to arginine. Lastly, amino acid uptake is also complemented by the action of IL4I1, an enzyme with a more ambiguous role in amino acid metabolism that we found highly expressed in ETs (FCH = 4.29). IL4I1 seems to contribute to the modulation of T cell responses by catalyzing the breakdown of local amino acids (phenylalanine) necessary for T cell activation, differentiation, and function, thus contributing to immune tolerance and anti-inflammatory responses [18]. Taken together, we observed that the proteomes of ETs and DTs reflected the different metabolic statuses of the T cells from different skin layers.

The pathway enrichment analysis highlighted differences in complement system regulation between ETs and DTs. We found elevated levels of multiple complement proteins (C3, C4a, CFD, CFB, C8, and C9) and regulatory proteins (CD59, vitronectin, and C4BPa) in ETs (Supplementary Figure S1d), while no difference was noted for C5, C7, CD46, and CD55 between ETs and DTs. C2 was detected exclusively in the ET samples. These findings support previous research showing T cells produce complement proteins independently of serum.

We found significantly higher expression of eight SERPINs in ETs, including the complement activation inhibitor SERPING1, the keratinocyte apoptosis inhibitor SERPINB3, and the skin inflammation-associated SERPINB4/B5 (Supplementary Figure S1d). In contrast, DTs showed significantly higher expression of six SERPINs: SERPINA1/A3/B1/B6 (protease inhibitors) and SERPINB8/B9 (with B9 inhibiting granzyme B). Overall, these patterns reveal distinct complement and SERPIN regulation between the two T cell populations.

### 2.3. MHC Molecules and Lipid Antigen Receptors in Epidermal and Dermal T Cells

**Figure 3:**
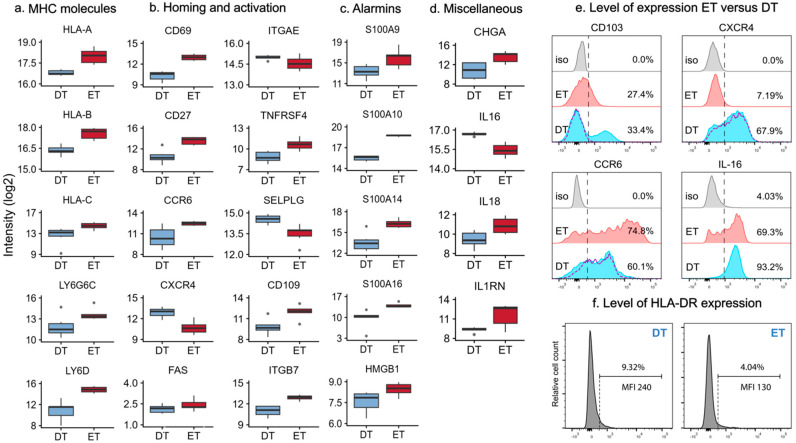
**Selected differentially expressed proteins in epidermal and dermal T cells.** Boxplots of ETs and DTs showing (**a**) MHC class I and MHC class II proteins, (**b**) homing and activation markers, (**c**) alarmins, and (**d**) CHGA, IL16, and IL18. Boxplots are based on imputed, log-2-transformed, filtered data (for proteins, minimum quantified in at least two-thirds of samples in at least one group). (**e**) Flow cytometric characterization of CD103, CCR6, CXCR4, and intracellular IL-16 in freshly isolated non-stimulated CD45+CD3+ cells, purified from epidermal (red) and dermal (blue) cell suspensions derived from normal human skin. ETs: epidermal T cells; DTs: dermal T cells; iso: isotype control (gray). Purple dashed line in DTs shows expression of indicated marker after treatment of DTs with trypsin. Numbers in histograms indicate percentages of positive cells. Data are representative of two independent experiments. (**f**) Estimation of HLA-DR expression in DTs and ETs, using original FACS data acquired during T cell isolation. MFI: mean fluorescence intensity. Data are representative of three and six independent experiments, respectively.

We observed significantly higher intensities of the classical MHC class I proteins HLA-A, HLA-B, and HLA-C in the epidermal T cell samples (FCH = 2.29, 2.33, and 3.57; Figure 3a and Supplementary Table S3). No difference was found in B2M, the essential stabilizing component of MHC class I molecules. In contrast, MHC class II molecules, such as HLA-DRA and HLA-DRB1, were quantified with higher intensities in the dermal T cell samples (FCH = 5.48 and 1.84, respectively). These molecules have an important role in antigen presentation, facilitating the activation and differentiation of T cells. The detection of HLA-DR in the T cell proteomes was paradoxical, given that HLA-DR was used as a negative lineage marker during FACS sorting. The signal in the proteomes was likely derived from intracellular protein pools rather than surface expression, reflecting a fundamental difference between the two techniques (i.e., surface proteins were detected during FACS sorting, whereas the proteomic approach in this study captured proteins from all cellular compartments). This interpretation aligns with a previous report demonstrating intracellular HLA-DR in T cells [19]. We observed elevated expression of caveolin-1 (CAV1: FCH = 4.51) in DTs, a major structural protein of caveolae, which are invaginations of the plasma membrane. CAV1 regulates T cell receptor (TCR) signaling and activation, participating in the costimulatory signals essential for TCR-mediated T cell activation [20]. We also noted higher intensities of MHC class III molecules LY6G6C (FCH = 3.36) and Ly6D (FCH = 15.6) (Figure 3a and Supplementary Table S3) in the ET cell compartment. Their genes are located on chromosome 6 and are highly conserved and remain functionally poorly characterized in the human system [21]. The lipid antigen receptors CD1a, CD1b, and CD1c were quantified only in a subset of the ET and DT samples, without notable differences between the groups (Supplementary Table S3).

### 2.4. Differentially Expressed Proteins Involved in Homing and Activation

Immune responses in the skin require tissue immunosurveillance and intercellular interactions between T cells and other cells in the skin. These activities are dependent on adhesion proteins, such as integrins, selectins, and cadherins. The skin-resident T cells often display a memory phenotype and typically express homing marker CD69, and a variable level of integrin CD103 (αE/ITGAE), which, together with ITGB7, can bind to E-cadherin on keratinocytes and, thus, contribute to tissue retention. We observed a higher intensity of CD69 (FCH = 6.41) and ITGB7 (FCH = 3.73) in ETs and similar intensities of ITGAE between groups (Figure 3b). Similarly, ETs had a higher intensity of the co-stimulatory molecules CD27 (FCH = 8.43) and OX40 (TNFRSF4 and CD134: FCH = 3.53) and inhibitory CD101 (FCH = 2.40). We found no difference in the co-stimulatory molecules GITR, CD2, and CD226 or the co-inhibitory protein CTLA. We observed differential expression of the homing molecules CCR6, SELPLG, and CXCR4 between the groups (Figure 3b and Supplementary Table S3). While ET cells had a higher expression of CCR6 (FCH = 4.06), in line with previous observations, DT cells had a higher intensity of SELPLG (FCH = 2.07), an established protein scaffold for CLA that facilitates trans-endothelial migration, and chemokine receptor CXCR4 (FCH = 4.55), which is involved in the chemotaxis and migration of T cells [22,23,24]. Both T cell groups expressed integrin ITGAL (alpha L) and ITGB2, needed for LFA-1 assembly. Similarly, both cell groups expressed ITGB1 but not ITGA4, needed for VLA-4 assembly. We also noted several molecules involved in cell signaling, such as CD109 and FAS (CD95). Finally, the S100 family of proteins, known for their antimicrobial functions, was represented with significantly higher intensities of S100A9, S100A10, S100A14, and S100A16 in the ETs (FCH = 6.18, 9.53, 6.48, and 21.1), as well as the alarmin HMGB1 (FCH = 1.85; Figure 3c and Supplementary Table S3). These results indicate that T cells in the epidermis and dermis are surrounded by distinct environments that result in clear differences in the levels of proteins involved in homing and T cell activation.

We also noted several proteins that are less characterized in the context of skin-derived T cells. One example is chromogranin A (CHGA), which is a marker of neuroendocrine tumors. Its functions in the skin might be linked to neuroendocrine–immune interactions and antimicrobial properties, and may explain the presence of CHGA in both ETs and DTs, with significantly higher expression in ETs [25].

In DTs, we found higher expression of IL-16 (FCH = 2.37; Figure 3d and Supplementary Table S2). IL-16 is a cytokine with pro-inflammatory effects when cleaved by caspase 3 [26]. It is expressed by several immune cells in the dermis, including T cells, especially CD4+ T cells [27]. In contrast with IL-16, IL-18 and IL4RN were only identified in ETs (Supplementary Table S2). IL-18 is a proinflammatory IL-1 family cytokine that is increasingly associated with atopic dermatitis [28]. IL4RN inhibits the activity of IL-1α and IL-1β and has a critical function in dampening inflammatory responses. Flow cytometry analysis of CCR6, CXCR4, and IL-16 expression largely validated the results obtained from mass spectrometry-based proteomics (Figure 3e).

The MS analysis revealed that a subset of dermal T cells expressed CD103 (Figure 3b), consistent with the presence of tissue-resident memory (TRM) T cells, previously reported by Watanabe et al. (2015) [3]. Unexpectedly, ETs showed lower CD103 expression, contradicting these earlier findings. This discrepancy led us to hypothesize that CD103 might be sensitive to the enzymatic digestion of the epidermis. We performed flow cytometry of trypsin-treated dermal T cells, which completely abolished CD103 surface expression (Figure 3e, purple dashed line), while other markers, including CXCR4 and CCR6, remained detectable, indicating marker-specific trypsin sensitivity. Notably, Watanabe et al. (2015) employed dispase rather than trypsin to generate single-cell suspensions from epidermal sheets, likely explaining the preserved CD103 expression in their study [3].

We addressed the potential concern that circulating T cells from dermal microvessels might have contaminated the dermal T cell (DT) population and contributed to the observed differential protein expression. Using B cells as a surrogate marker for blood contamination, given their high prevalence in blood and scarcity in healthy skin, we demonstrated that CD19^+^ B cells were virtually undetectable in dermal samples (Supplementary Figure S2a,b). Based on the B cell-to-T cell ratio in blood, we estimate that less than 0.5% of T cells in our dermal suspensions could be blood-derived. Since low frequencies of skin-resident B cells have been reported in normal human skin by immunohistochemistry, this estimate likely represents an upper limit, further confirming minimal blood contamination [29].

To verify the HLA-DR data, we revisited the original FACS data acquired during T cell isolation. As shown in Figure 3f, only a small subset of cutaneous T cells expressed HLA-DR, with 11.1 ± 1.6% of dermal T cells and 7.5 ± 2.7% of epidermal T cells showing HLA-DR positivity at very low levels (MFI 274 and MFI 195, respectively). It is important to note that these HLA-DR+ T cells were excluded from our proteomic analysis due to the applied sorting strategy.

Collectively, our proteomic analysis reveals distinct protein signatures between epidermal and dermal T cells, highlighting compartment-specific expression patterns of adhesion molecules, activation markers, and cytokines, while also uncovering novel proteins like CHGA and IL-16 that may play previously unrecognized roles in skin immune responses.

## 3. Discussion

Our study provides an in-depth proteomic characterization of skin-derived epidermal and dermal T cells. We quantified 5985 proteins in total, with a significant overlap (93.5%) in protein expression between both groups. The recent technological advances in mass spectrometry enabled us to identify 57% more proteins per sample than our previously published data on DTs and ETs [9]. While our data suggest that ETs and DTs share many functions, we identified over 2000 proteins that were differentially expressed between the cells from the two skin layers, pointing to potential distinct functional roles reflective of the anatomical positioning and varying immune challenges these cells face. However, given the experimental design and methodological constraints, these findings should be viewed as exploratory rather than definitive. The distinct molecular signatures between ETs and DTs were especially evident in their complement system components, metabolism, and immune regulatory molecules.

A key finding was the elevated expression of complement proteins (e.g., C3, C4a, factors B and D, and membrane attack complexes) in ETs. This localized complement system may represent a defense mechanism, independent of serum-derived complement proteins, which could support rapid responses to pathogens on the skin’s outer barrier. Previous studies have demonstrated that T cells can produce complement proteins and function independently of serum-derived complement proteins [30], but our findings suggest compartment-specific differences even between T cells in the same tissue. The concurrent expression of complement regulatory proteins (CD59, vitronectin, and C4BPa) suggests a balanced system that can mount effective responses while preventing excessive inflammation [31].

Another surprising finding was the distinct metabolic signatures between ETs and DTs. The elevated levels of proteins linked to OXPHOS in ETs, coupled with an elevated PDK1 expression (an enzyme that inhibits pyruvate dehydrogenase and potentially reduces pyruvate entry into the TCA cycle), suggest a proteomic signature consistent with a preference for OXPHOS over glycolysis in ETs, rather than definitive evidence of metabolic rewiring. This metabolic programming aligns with previous reports on tissue-resident memory T cells, including experiments that suggest a substantial spare respiratory capacity in their mitochondria that is useful during activation [8,32,33]. Additionally, we observed differences in lipid and cholesterol metabolism, with increased levels of key cholesterol biosynthesis enzymes DHCR24 and DHCR7, along with elevated expression of apolipoproteins (APOC2, APOC3, APOE, and APOH) and the cholesterol transport protein TSPO in ETs. These findings suggest enhanced cholesterol metabolism, which may reflect adaptation to the cholesterol-rich epidermal environment [34]. Given the essential role of cholesterol in lipid raft formation and T cell receptor signaling, functional validation is required to determine whether these proteomic differences contribute to a specialized membrane organization that supports tissue-resident T cell function in situ [35]. Furthermore, the differential cholesterol metabolism between ETs and DTs may influence their long-term survival and function. Tissue-resident memory T cells are known to rely on fatty acid metabolism for their maintenance, preferentially using fatty acid synthesis to fuel mitochondrial fatty acid oxidation, and cholesterol metabolism is increasingly recognized as a critical regulator of T cell function and longevity [8,36,37]. Hence, the enhanced cholesterol biosynthetic capacity of ETs, coupled with their elevated OXPHOS machinery, suggests a dual-edged metabolic program optimized for both long-term tissue residence and rapid response capability. In addition, the differences in cholesterol metabolism could also account for the previously reported lower motility of T cells in the epidermis [38]. While these metabolic features are compelling, we emphasize that our findings should be viewed as hypothesis-generating rather than conclusive, and future functional studies will be required to validate these pathways and their relevance to tissue residency and immune function.

The differential expression of amino acid transporters and metabolic enzymes, particularly the marked increase in SLC7A5 and ASS1 in ETs, points to distinct nutrient utilization strategies that could be coupled to T cell function and CD69 expression [39]. CD69, an established tissue-residency marker highly expressed in ETs, has been mechanistically linked to amino acid transport through the LAT1/mTOR signaling axis [40]. This connection may explain our finding of elevated mTOR and SLC7A5 (LAT1) levels in ETs, suggesting a coordinated program supporting tissue residence and metabolic adaptation. The marked upregulation of ASS1 in ETs points to a specialized arginine metabolism program. Arginine metabolism has emerged as a critical regulator of T cell function and longevity, with recent studies demonstrating its ability to suppress glycolysis while maintaining mitochondrial function [10,41]. This metabolic profile further supports our finding of enhanced OXPHOS machinery in ETs, suggesting that arginine metabolism helps maintain their tissue-resident phenotype while preserving their capacity for rapid immune responses.

Our proteomic profiling of tissue residency-associated molecules, such as ITGAE, ITGA1 (CD49a), CD101, CD44, CXCR4, AHR, and FABP5, confirmed well-documented findings in the literature [42,43]. The higher expression of CD69 in ETs supports their resident memory phenotype and survival capacity, consistent with previous studies on tissue-resident memory T cells [44], although here, the data suggest differences in the expression between skin layers as well. The elevated CD27 expression in ETs is a surprising finding given that this co-stimulatory molecule is typically characteristic of naïve T cells rather than memory populations. However, Rieckman et al. (2017) demonstrated CD27 expression at both the protein and RNA levels in central and effector memory T cells, both under steady-state conditions and following activation [11]. These findings suggest that CD27 expression on memory T cells may be more context-dependent than previously assumed [45]. The expression of chemokine receptors CCR6 and CXCR4 in skin-resident T cells is well documented, although the differential expression between the layers adds a new detail [46]. The epidermal T cells expressed higher intensities of MHC class I molecules, whereas dermal T cells expressed higher levels of MHC class II molecules, suggesting compartmental distinctions in antigen presentation mechanisms.

Our proteomic datasets represent one of the most comprehensive descriptions of the skin T cell proteomes and indicate that the proteome composition of T cells varies depending on their skin layer. Although robust, our study does have limitations that warrant acknowledgment. Firstly, we used a pooled sample approach, which may mask minor specific subpopulation characteristics, including resident memory T cells, MAIT cells, Treg cells, and γδ T cells, each of which may be differentially distributed between the epidermis and dermis [4,5,6]. Furthermore, the lack of paired epidermal and dermal T cells from the same donors, as well as the absence of CD4/CD8 subdivision within the epidermal compartment, may have limited additional functional insights. Secondly, mass spectrometry-based proteomics has inherent limitations in quantifying low-abundance cytokines and certain signaling molecules. Moreover, our proteomic data do not provide information on the cellular compartment of origin, as it detects proteins from all cellular compartments, including surface, cytoplasmic, and nuclear fractions. This contrasts with standard FACS analysis, which selectively measures cell surface proteins on non-permeabilized cells. This fundamental difference likely explains the paradoxical detection of HLA-DR in our proteomic dataset, despite having excluded HLA-DR^+^ T cells during FACS sorting. The HLA-DR signal observed by mass spectrometry is most likely derived from intracellular compartments, rather than surface expression. Thirdly, epidermal and dermal T cells are isolated using different enzymatic dissociation methods (trypsin versus collagenase) due to the distinct compositions of the two skin compartments, which may yield unexpected outcomes. Trypsin is a relatively potent protease, whereas collagenase primarily targets collagen fibers to release dermal cells without affecting most surface proteins. The loss of trypsin-sensitive cell surface markers, as demonstrated for CD103, is an important factor to consider when comparing the two compartments. Lastly, T cells isolated from the dermis may include contaminating peripheral T cells from the cutaneous microvasculature. These methodological factors could significantly influence protein abundance profiles and should temper overinterpretation of observed differences. Therefore, our results are best interpreted as an exploratory proteomic map that generates hypotheses for future targeted investigations.

In conclusion, our comprehensive proteomic analysis reveals putative specializations of epidermal and dermal T cells, highlighting their unique contributions to skin immunity. These findings provide proteomic evidence suggestive of functional divergence but require further experimental validation. Our study contributes to a foundation for future research aimed at targeting specific T cell populations to improve therapeutic strategies for skin-related diseases.

## 4. Materials and Methods

### 4.1. Samples and Cell Isolation

The in-depth mass spectrometry-based re-analyses presented in this study are based on our original raw MS files that were made available as part of the Human Skin Proteome Atlas [9]. Residual human skin tissue was obtained from healthy donors (aged between 18 and 65 years) undergoing corrective surgery of the breast or abdomen. Skin sheets were prepared with an electrodermatome and treated overnight at 4 °C with 0.2% (*wt*/*vol*) dispase II (Boehringer Mannheim, Mannheim, Germany) in PBS with 50 µg/mL gentamicin (Sigma, St. Louis, MO, USA). After separation of the epidermis and dermis by forceps, the epidermis was enzymatically digested with 0.25% trypsin in PBS for 30 min at 37 °C, while the dermis was treated with 0.4% collagenase D in IMDM (Fisher Scientific, Leicestershire, UK). Digested tissue suspensions were sieved through 70 µm cell strainers, and subsequently, cells were labeled with fluorochrome-conjugated anti-human protein antibodies and sorted by FACSAria cell-sorting equipment (BD Biosciences, Franklin Lakes, NJ, USA).

To stain epidermal cells we used (dilution, clone, catalog number) APC-Cy7-conjugated CD45 (1:200, 2D1, 348815), PE-CF594-conjugated CD3 (1:200, UCHT1, 562280), PE-conjugated HLA-DR (1:100, G46-6, 555812), FITC-conjugated CD1a (1:100, HI149, 555806) from BD Biosciences, and APC-conjugated CD94 (1:100, DX22, 305508) from BioLegend, San Diego, CA, USA. To stain dermal cells, we used APC-Cy7-conjugated CD45, PE-conjugated HLA-DR, FITC-conjugated CD14 (1:100, MφP-9, 345784), FITC-conjugated CD94 (1:100, HP-3D9, 555888), APC-conjugated CD11c (1:100, S-HCL-3, 333144), PE-Cy7-conjugated CD8 (1:100, RPA-T8, 557746) from BD Biosciences, and BV421-conjugated FcεRIa (1:100, AER-37, 334624), PE-Dazzle594–conjugated CD4 (1:200, OKT4, 317448), BV605–conjugated CD3 (1:100, OKT3, 317322) from BioLegend. DTs were identified by their CD45+CD3+ expression profile and further refined through exclusion of cells expressing HLA-DR, FcεR, CD1a, CD11a, CD14, or CD94. Similarly, ETs (CD45+CD3+) were isolated following exclusion of HLA-DR, CD1a, and CD94-expressing populations. Sorted cells were washed twice with PBS, snap-frozen in liquid nitrogen, and stored at −80 °C until sample preparation for LC-MS/MS. A detailed methods section can be found in Dyring-Andersen et al. (2020) [9].

For the experiments presented in Figure 3e, we isolated dermal and epidermal T cells from residual human skin tissue (N = 2) from healthy donors, using the same method as described above and the following fluorochrome-conjugated anti-human protein antibodies: CD103–PE-Cy7 (1:100, Ber-ACT8, 350212), CCR6–BV421 (1:100, G034E3, 353408), and IL-16–PE (1:100, 14.1, 519106) from BioLegend, and CXCR4–APC (1:100, 12G5, 306510) from eBioscience (San Diego, CA, USA). For the experiments presented in Supplementary Figure S2a, we used CD19–PE (1:100, HIB19, 302208) from BioLegend and CD3–PE-CF594 from BD Biosciences. Flow cytometry was performed with an LSRFortessa (BD Biosciences), and analysis was performed with the FlowJo software (TreeStar, Ashland, OR, USA). 

### 4.2. Ethics Statement

This study was carried out in agreement with the Danish and Dutch law (Medical Research Involving Human Subjects Act), in accordance with the guidelines of the Medical Ethics Review Committees of the involved institutes, and following the Declaration of Helsinki principles.

Human peripheral blood was obtained from healthy adult volunteers after written informed consent was obtained using an approved protocol by the Ethics Committee of the Capital Region of Denmark (H-3-2014-123). Residual skin tissue from elective corrective surgery (breast or abdominal procedures) was obtained from hospitals in both Denmark and the Netherlands. These tissue samples were anonymized prior to being provided to the researchers. In accordance with both Danish and Dutch regulations, fully anonymized residual tissue from standard medical procedures may be used for scientific research without written informed consent or additional approval by a medical ethics committee, provided that patients are properly informed via their attending physician that their tissue may be used for research, and that they have not objected.

No personal or identifiable data were accessed or processed by the researchers, and all procedures complied with the principles of data protection, as outlined in the EU General Data Protection Regulation.

### 4.3. LC-MS/MS and Data Analysis

Data-independent acquisition (DIA) raw files were reanalyzed using the Spectronaut Pulsar software (Biognosys, version 18, Biognosys, Switzerland) and default settings for targeted DIA analysis with the ‘mutated’ as decoy method. The data export was filtered by ‘No Decoy’ and ‘Quantification Data Filtering’ for peptide and protein quantifications.

We also noted multiple structural proteins in the T cells associated with their compartmental origin, but no difference between groups in the percentage of summed protein intensities that could skew the intensities of other less abundant proteins in the groups.

### 4.4. Statistical Analysis

All statistical and bioinformatic analyses were performed using Perseus (version 1.6.5) [47,48]. Data were filtered for proteins that were present in at least 60% of the samples in one group and normalized using log-2 transformation. Missing values were imputed using the MinProb approach (with random draws from a Gaussian distribution; width = 0.3 standard deviations (SDs) and downshift = 1.8 SD) before statistical testing. Differential expression analysis was conducted using an unpaired Student’s *t*-test for ETs vs. DTs. A permutation-based false discovery rate (FDR) of 5% was applied, with proteins having a q-value below 0.05 considered to be significantly differentially expressed.

To investigate the presence of transcription factors in our data, we used the Molecular Signatures Database (MSigDB) [49,50].

## Data Availability

The proteomics raw data and quantified files were submitted to the ProteomeXchange Consortium through the PRIDE partner repository (https://www.ebi.ac.uk/pride/, accessed on 15 July 2025) with the identifier PXD019909. The data are also available in the Supplementary Tables and at http://skin.science, accessed on 15 July 2025.

## Supplementary Materials

The following supporting information can be downloaded at https://www.mdpi.com/article/10.3390/ijms26167942/s1.
