# Supplementary material for: Epidermal and Dermal T Cells Exhibit Distinct Proteomic Signatures
Source: Int J Mol Sci. 2025 Aug 18;26(16):7942. doi: 10.3390/ijms26167942 (PMC12386419; doi:10.3390/ijms26167942)
Supplement: Supplementary file 1 [file ijms-26-07942-s001.zip › ijms-3741786 Supplementary Figure.pdf]

Supplementary Figure S1. Flow cytometric sorting strategy, T cell associated molecules and complement proteins in ET and DT

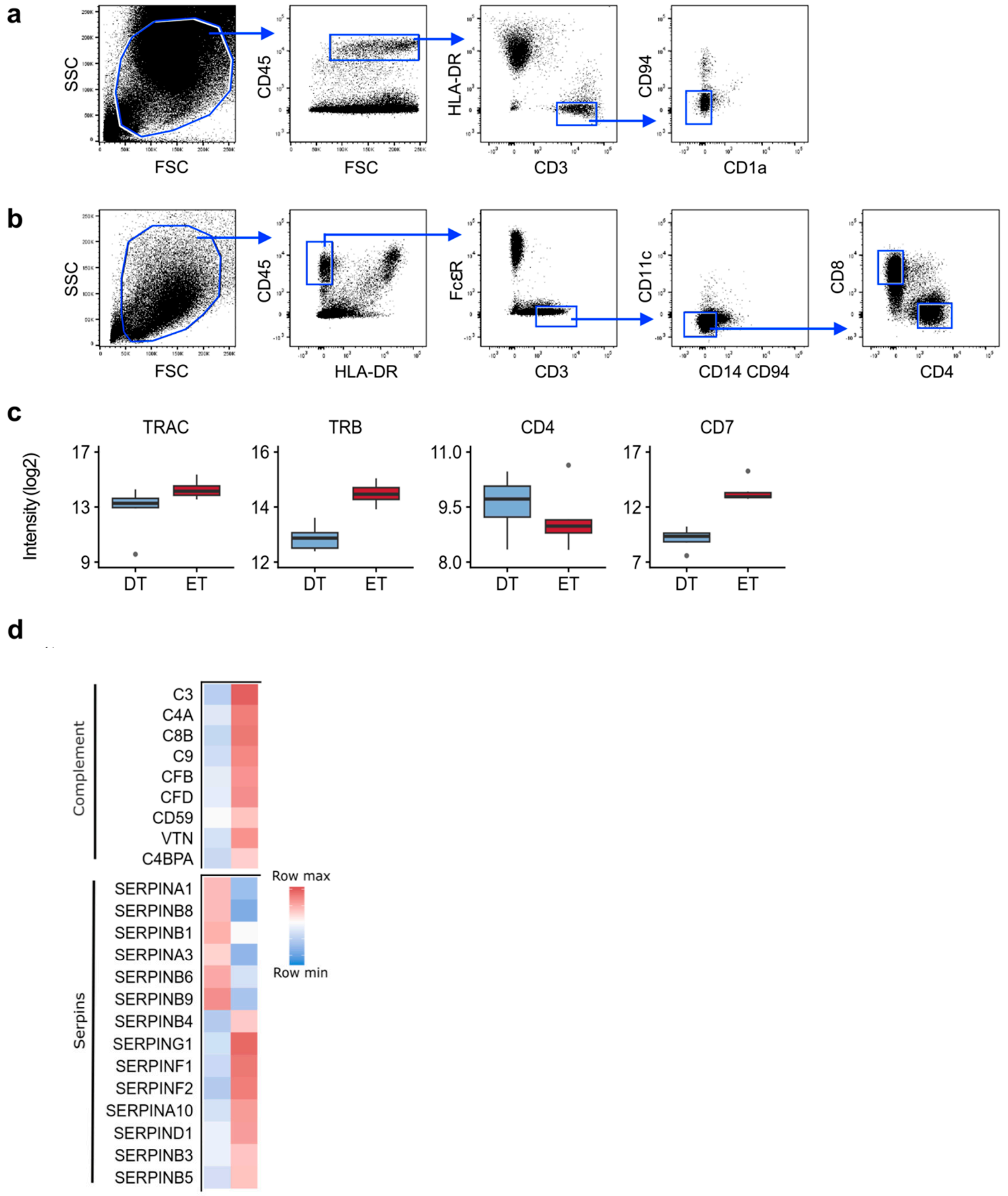

Supplementary Figure S1. Flow cytometric sorting strategy, T cell associated molecules and complement proteins in ET and DT

Flow cytometry sorting strategy for the isolation of ET and DT. (a) ET were isolated as CD45+CD3+ cells which lacked HLA-DR, CD1a and CD94. (b) The CD45+CD3+ dermal T cells were subdivided into CD4+ and CD8+ T cells after application of the exclusion markers HLA-DR, FcεR, CD1a, CD11c, CD14 and CD94. (c) Boxplots of log2 transformed, raw intensity quantifications of alpha chain (TRAC) and beta chain (TRB) of the TCR, CD4, CD8a and CD7 in ET and DT. (d) Heatmaps of the complement proteins and SEPRINs. The heatmaps are colored by row Z-score and based on imputed, filtered data (for proteins minimum quantified in at least two-thirds of samples in at least one group).

Supplementary Figure S2

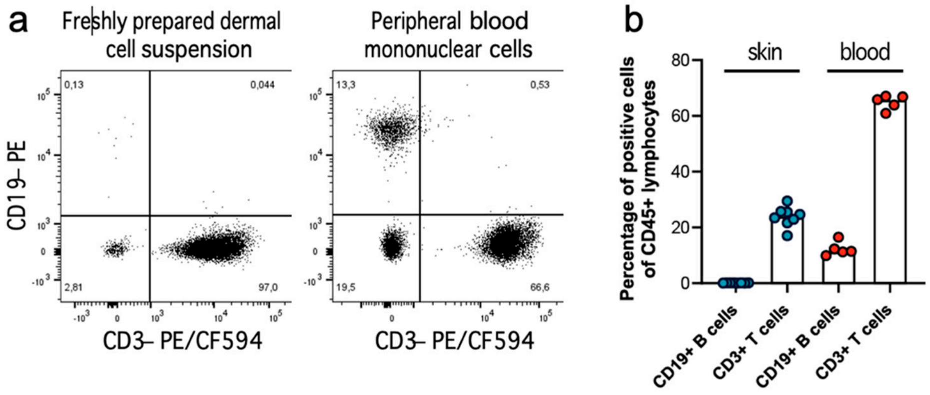

Supplementary Figure S2. Flow cytometry of CD3 and CD19 cells in dermal cell suspensions and peripheral blood. (a) Representative flow cytometry plots of dermal cells (left, from healthy skin), stained for CD3, CD19, and CD45. Plots show live CD45$^+$ lymphocytes. (b) Quantification of the proportion of B cells and T cells in healthy skin (blue dots, N=8) and blood (red dots, n=5) expressed as percentage of CD45$^+$ lymphocytes. The mean frequencies of CD19$^+$ B cells and CD3$^+$ T cells were 0.07 and 23.8 (yielding a B:T ratio of 1:340), while the respective values in blood were 10.6 and 69.2 (B:T ratio of 1:6.5).
